# A Water-Soluble Small Molecule Boron Carrier Targeting Biotin Receptors for Neutron Capture Therapy

**DOI:** 10.1021/acsomega.4c09388

**Published:** 2024-12-18

**Authors:** Kai Nishimura, Shota Tanaka, Kazuki Miura, Satoshi Okada, Minoru Suzuki, Hiroyuki Nakamura

**Affiliations:** † School of Life Science and Technology, Institute of Science Tokyo, 4259 Nagatsuta-cho, Midori-ku, Yokohama 226-8501, Japan; ‡ Laboratory for Chemistry and Life Science, Institute of Integrated Research, Institute of Science Tokyo, 4259 Nagatsuta-cho, Midori-ku, Yokohama 226-8501, Japan; § Institute for Integrated Radiation and Nuclear Science, Kyoto University, 2-1010, Asashiro-Nishi, Kumatori-cho, Sennan-gun, Osaka 590-0494, Japan

## Abstract

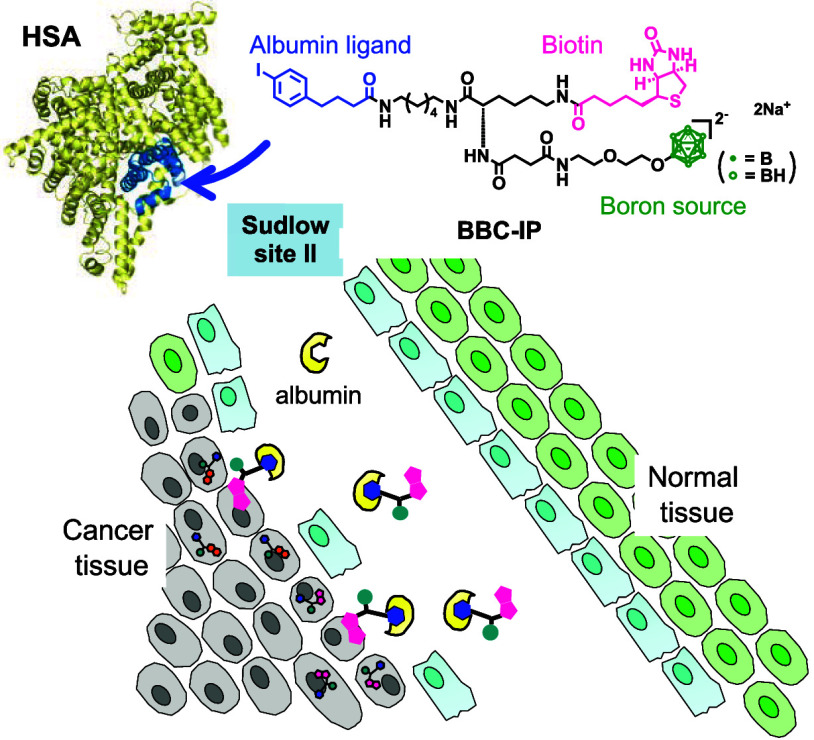

A critical challenge in boron neutron capture therapy (BNCT) is expanding its effectiveness through the development of novel boron agents with different mechanisms of action than the approved drug 4-borono-l-phenylalanine (BPA). In this study, we developed a small molecule boron carrier, biotinyl-*closo*-dodecaborate conjugate with an iodophenyl moiety (BBC-IP), incorporating biotin as a ligand for biotin receptors overexpressed in various cancer cells, alongside an albumin ligand and boron source. BBC-IP exhibited high water solubility, minimal cytotoxicity, and superior cellular uptake compared to BPA in both human and mouse cancer cells. Biodistribution studies revealed that BBC-IP achieved enhanced tumor accumulation (9.7 μg [B]/g, 3 h) in mouse colon tumors, surpassing BPA’s accumulation levels (7.2 μg [B]/g, 3 h) at a dose of 15 mg [B]/kg. However, despite this improved tumor accumulation, BPA demonstrated superior BNCT efficacy. The intracellular localization of boron agents in tumor cells revealed that BPA localized throughout the cell, whereas BBC-IP localized mainly in the cytoplasm. These results indicate the intratumoral localization, as well as tumor accumulation are critical for the efficacy of novel BNCT agents.

## Introduction

Boron neutron capture therapy (BNCT) is a noninvasive cancer particle beam therapy utilizing a nuclear reaction involving the boron isotope (^10^B) and thermal neutrons.^1^ The resulting highly cytotoxic α-particles (^4^He) and ^7^Li nuclei have a short-range of 4–10 μm, approximately equivalent to the diameter of a single cell. This property allows the BNCT to selectively target tumor cells by accumulating ^10^B-containing boron drugs, thereby enabling the targeted destruction of cancer cells while minimizing damage to healthy cells. 4-Borono-l-phenylalanine (BPA) is the most widely used boron agent in clinical BNCT to date. It gained approval and insurance coverage in Japan under the name Borofalan (Steboronine) in 2020 for the treatment of unresectable, locally advanced, or locally recurrent head and neck cancers.^2^ Borofalan, mimicking phenylalanine, is actively transported into tumor cells thorough L-type amino acid transporter 1 (LAT-1), which is overexpressed in various cancer cells, including glioma.^3^ However, several cancer types with low LAT-1 expression levels have been reported, limiting the applicability of BPA-based BNCT.^4^ Hence, there is an urgent need to develop novel boron carriers with distinct tumor-uptake mechanisms to expand the range of BNCT applications. Various boron carriers have been devised to be incorporated via mechanisms distinct from LAT-1, addressing this critical need for expanded BNCT efficacy.^5^

**Figure 1:**
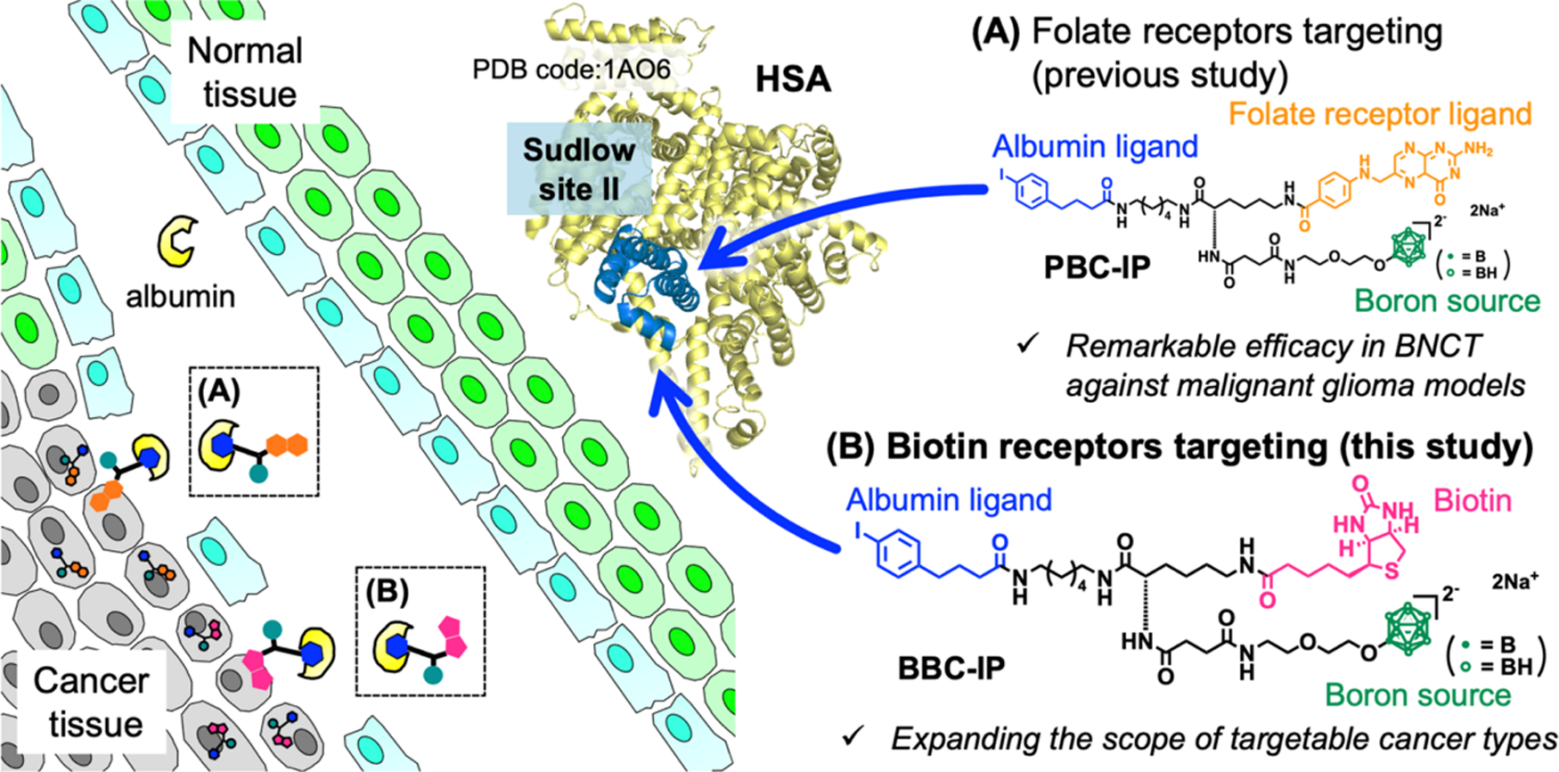
Boron delivery systems targeting tumor cells via endogenous albumin and tumor-specific proteins for BNCT. (A) PBC-IP (previous work): Targeting folate receptors. (B) BBC-IP (this work): Targeting biotin receptors.

We have focused on the tumor-selective accumulation of albumin via the long-term blood retention and the enhanced permeability and retention (EPR) effect, resulting in the development of a boron delivery system that utilizes albumin for targeted delivery to tumors.^6−9^ Recently, we have successfully developed a small molecule boron carrier, pteroyl-*closo*-dodecaborate conjugated with a 4-(*p*-iodophenyl)butyric acid moiety (PBC-IP, Figure 1A), which not only utilizes endogenous albumin but also targets folate receptors overexpressed on the surfaces of various cancer cells.^10^ PBC-IP has demonstrated remarkable efficacy in BNCT against malignant gliomas with low BPA accumulation in animal models, and preclinical studies of PBC-IP are currently ongoing.

Biotin is widely recognized as a crucial vitamin essential for the proliferation of all cells, particularly playing a significant role in the growth of tumor cells. Biotin receptors (BRs) are known to be overexpressed in a variety of cancer cells,^11^ including those with low folate uptake^12^ and breast cancer cells that are suitable candidates for BNCT.^13^ Among BRs, the Sodium-Dependent Multivitamin Transporter (SMVT) is implicated in biotin uptake and is considered a potential cancer biomarker for tumor diagnosis.^14^ While some proteins within BRs remain undiscovered, strategies targeting BRs show substantial promise and could lead to the development of anticancer drugs such as biotinylated Gemcitabine,^15^ SN-38,^16^ paclitaxel,^17^ and cisplatin.^18^ Regarding biotinylated boron-containing small molecules, only a biotinylated phenylboronic acid^19^ and magnetic resonance imaging (MRI)/Gd BNCT theranostic contrast agent^20^ have been reported. However, the former is not intended for use in BNCT, and the latter, using a poorly water-soluble carborane as the boron source, remains untested in cellular or animal models. Biotin is water-soluble at approximately 0.9 mM, but decreases when its carbonyl group is employed to bind lipophilic functional groups. Given this context, the persistent challenge remains in formulating water-soluble biotinylated boron carriers designed specifically for BNCT.

In this study, we designed a biotinyl-*closo*-dodecaborate conjugate with an iodophenyl moiety (BBC-IP, Figure 1B) by converting the pteroyl group, which functions as a folate receptor ligand in PBC-IP, into biotin. BBC-IP shares similarities with PBC-IP, including an iodophenyl moiety acting as an albumin ligand that binds to Sudlow site II of human serum albumin (HSA),^21^ and containing a water-soluble boron cluster. We hypothesized that BBC-IP would enhance water solubility compared to biotin, interact with albumin, and exert biotin-mediated uptake on cancer cells that previously have shown that they do not internalize PBC-IP.

## Results and Discussion

### Chemistry

**Scheme 1:**
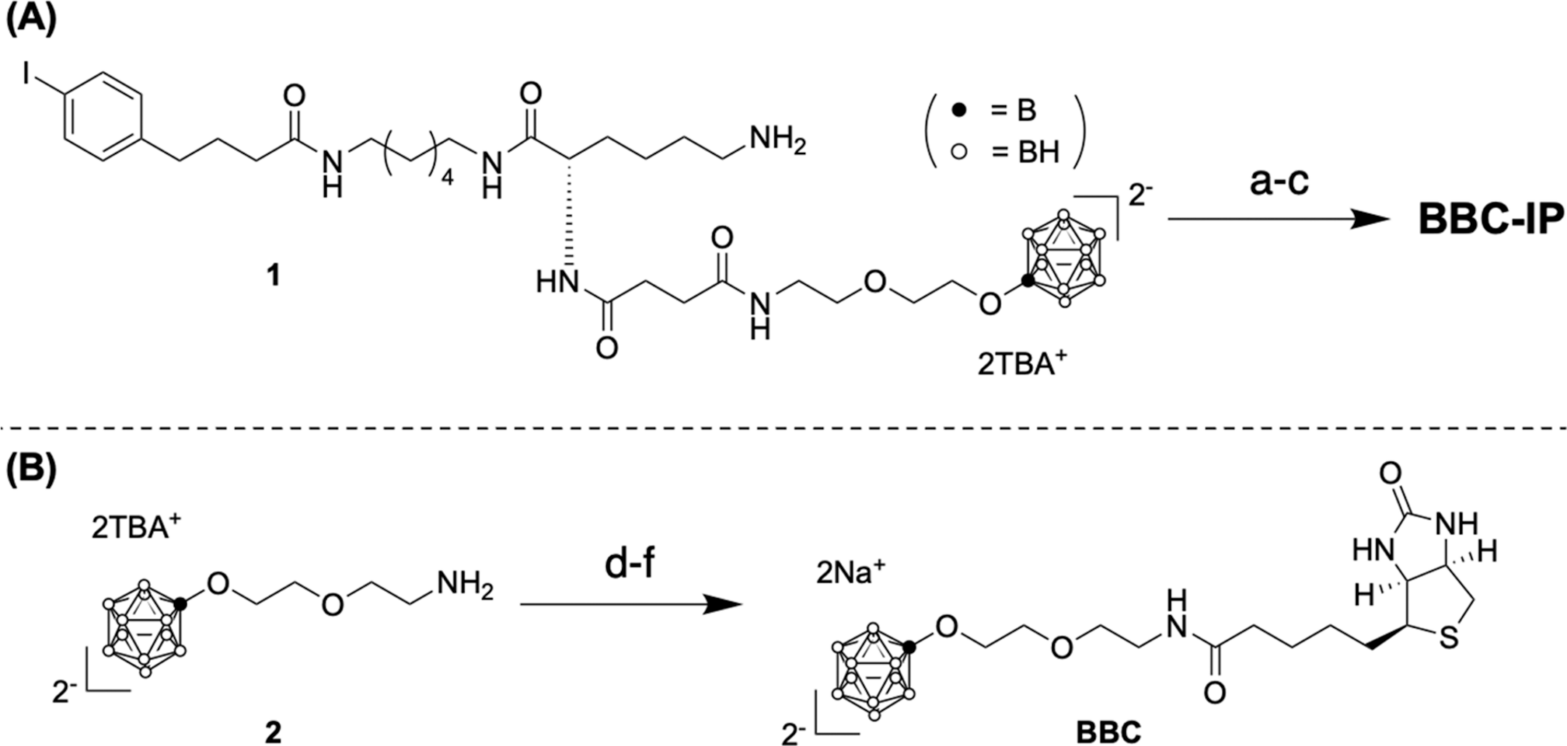
Synthesis Route of (A) BBC-IP and (B) BBC Reagents and conditions: (a) Biotin, EDCI·HCl, HOBt·H_2_O, DIEA, DMF, rt, 40 h; (b) TMACl, DMF, EtOH, rt, 1 h; (c) Na^+^-Amberlite, CH_3_CN, H_2_O, rt, 18 h, 89% (3 steps, BBC-IP); (d) Biotin, EDCI·HCl, HOBt·H_2_O, DIEA, DMF, rt, 14 h; (e) TMACl, DMF, EtOH, rt, 30 min; (f) Na^+^-Amberlite, H_2_O, rt, 16 h, 33% (3 steps, BBC). The abbreviations used in this reaction scheme: DIEA = *N*,*N*-diisopropylethylamine; DMF = *N*,*N*-dimethylformamide; EDCI = 1-(3-dimethylaminopropyl)-3-ethylcarbodiimide; HOBt = 1-hydroxybenzotriazole; TMA = Tetramethylammonium.

Figure 1 illustrates our strategy for boron delivery systems, comparing the previously established PBC-IP with the currently designed BBC-IP, in which the pteroyl unit of PBC-IP is replaced by biotin. Initially, we conjugated the iodophenyl moiety and tetrabutylammonium *closo*-dodecaborate onto a lysine scaffold based on our previous synthetic protocol of PBC-IP.^10^ Subsequent steps included the condensation of biotin with amine **1**, exchange of the countercation of *closo*-dodecaborate from tetrabutylammonium to sodium to enhance water solubility. This process afforded BBC-IP in 89% yield in three steps (Scheme 1A). Additionally, we synthesized a biotin-*closo*-dodecaborate conjugate (BBC) without the iodophenyl moiety for comparison. BBC was synthesized by condensation of biotin with *closo*-dodecaborate derivative **2**, followed by exchange of the countercation of the *closo*-dodecaborate with sodium, in 33% yield in three steps (Scheme 1B).

### Affinity of BBC-IP to HSA

**Figure 2:**
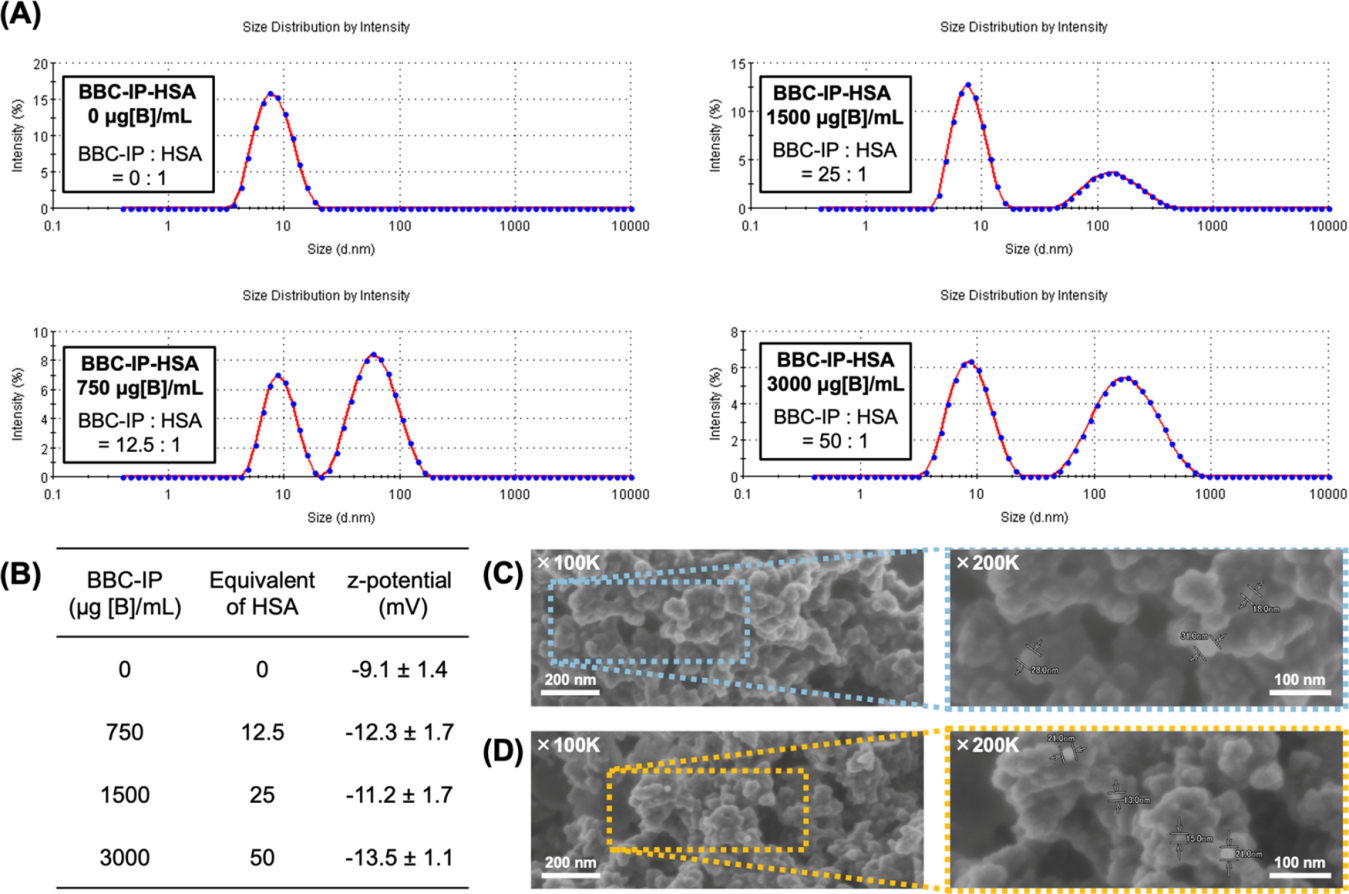
(A) Effect of BBC-IP concentration on HSA size distribution. The particle size distribution was measured by dynamic light scattering (DLS) analysis. The final concentration of HSA was 0.463 mM, while the final concentrations of BBC-IP were 0, 750, 1500, and 3000 μg [B]/mL, corresponding to 0, 12.5, 25, and 50 mol equiv of BBC-IP with respect to HSA, respectively. BBC-IP-HSA refers to the conjugate of BBC-IP and HSA. (B) ζ-potential analysis of the same samples in panel (A). Data are shown as the mean ± SD deviation of the triplicated measurements. (C, D) Field emission scanning electron microscopy (FE-SEM) images of (C) HSA and (D) BBC-IP-HSA.

As described in the Introduction, PBC-IP and BBC-IP were designed to target folate and biotin receptors overexpressed on cancer cell surfaces, with structural differences in their small-molecule ligands—pteroyl and biotin moieties, respectively. Both compounds contain an iodophenyl group that can serve as a noncovalent ligand for albumin. We initially evaluated the interaction between BBC-IP and HSA through an enzyme-linked immuno sorbent assay (ELISA). The dissociation constant (*K*_d_) of BBC-IP was estimated from semilogarithmic dose–response curves of its fluorescence intensity and determined to be 5.16 ± 1.09 mM. For comparison, the previously reported *K*_d_ value for PBC-IP was 11.7 ± 0.8 μM (Figure S1, Supporting Information). These results indicate that the biotin moiety of BBC-IP does not significantly contribute to its interaction with HSA compared to the pteroyl moiety of PBC-IP and may even hinder this interaction. Previous study has shown that biotin and folic acid exhibit similar interactions with albumin, binding to fatty acid binding site 1 (FA1) in subdomain 1B and FA5 in subdomain IIIB, with binding constant (*K*_b_) values of 4.12 × 10^4^ and 8.1 × 10^4^ M^–1^, respectively.^22,23^ We attributed the difference in binding affinity between BBC-IP and PBC-IP to two possibilities. First, BBC-IP lacks the carboxyl group that is conventionally present in biotin. Second, the biotin-binding site on albumin may not be optimally position for the iodophenyl group-binding site of Sudlow site II. Dynamic light scattering (DLS) analysis indicated that human serum albumin (HSA) in phosphate buffer solution (PBS) did not aggregate under the conditions tested (Figure 2A). Upon addition of BBC-IP, multimer formation was observed, with diameters increasing in a concentration-dependent manner, reaching peak values in the range of 60 to 200 nm. ζ-potential analysis showed that the addition of BBC-IP resulted in a more negative charge on albumin (Figure 2B). This effect is attributed to the ionic nature of BBC-IP, with the albumin ligand encapsulated within its anionic component. Field emission scanning electron microscopy (FE-SEM) analysis revealed no significant differences in the observations between HSA measured alone and the conjugate of BBC-IP and albumin (BBC-IP-HSA, Figure 2C,D).

### Stability Evaluation

Stability assessments were conducted by monitoring the UV–vis spectra of BBC-IP and BBC in PBS (simulating *in vitro* conditions) and in FBS (simulating *in vivo* conditions) at 37 °C. Over a period of 6 days, minimal changes were observed in both media, indicating that BBC-IP and BBC remained stable under conditions that mimic *in vitro* and *in vivo* environments (Figure S2, Supporting Information).

### Cytotoxicity, Cellular Uptake, and Intracellular Distribution

Boron agents used in BNCT require high water solubility and low toxicity. BPA used in the clinical treatment of BNCT has achieved these two properties by forming a complex with sorbitol. Upon evaluating the water solubility of BBC-IP and BBC, we observed that both compounds displayed considerable water solubility (>40 mM) without any additives. Subsequently, we assessed their cytotoxicity using the 3-(4,5-dimethyl-2-thiazolyl)-2,5-diphenyltetrazolium Bromide (MTT) assay on various cancer cell lines including A549 (human lung carcinoma), B16 (mouse melanoma), CT26 (mouse colon carcinoma), MDA-MB-231 (human breast adenocarcinoma), and U87MG (human glioblastoma) cells. Median inhibitory concentration (IC_50_) values of BBC-IP were estimated to be 0.96 ± 0.07, 0.17 ± 0.04, 0.26 ± 0.08, 0.15 ± 0.04, and 0.24 ± 0.01 mM for A549, B16, CT26, MDA-MB-231, and U87MG cells, respectively (Table 1). BBC and BPA showed no significant cytotoxicity at 1 mM, except for BPA with an IC_50_ of 0.91 ± 0.13 mM against B16 cells. These results indicate that BBC-IP and BBC are low molecular weight boron compounds with highly water-soluble and sufficiently low cytotoxic to meet the criteria as boron agents for BNCT.

**Table 1:** Cytotoxicity of BBC-IP, BBC, and BPA against Several Cancer Cell Lines

	cytotoxicity IC_50_ (mM)^a^^,^^b^		
cell line	BBC-IP	BBC	BPA
A549	0.96 ± 0.07	>1	>1
B16	0.17 ± 0.04	>1	0.91 ± 0.13
CT26	0.26 ± 0.08	>1	>1
MDA-MB-231	0.15 ± 0.04	>1	>1
U87MG	0.24 ± 0.01	>1	>1

a IC_50_ values are expressed as mean ± SEM from a single experiment conducted in triplicate.

b Cell proliferation was measured by the MTT assay against each cell line incubated with compounds for 72 h.

**Figure 3:**
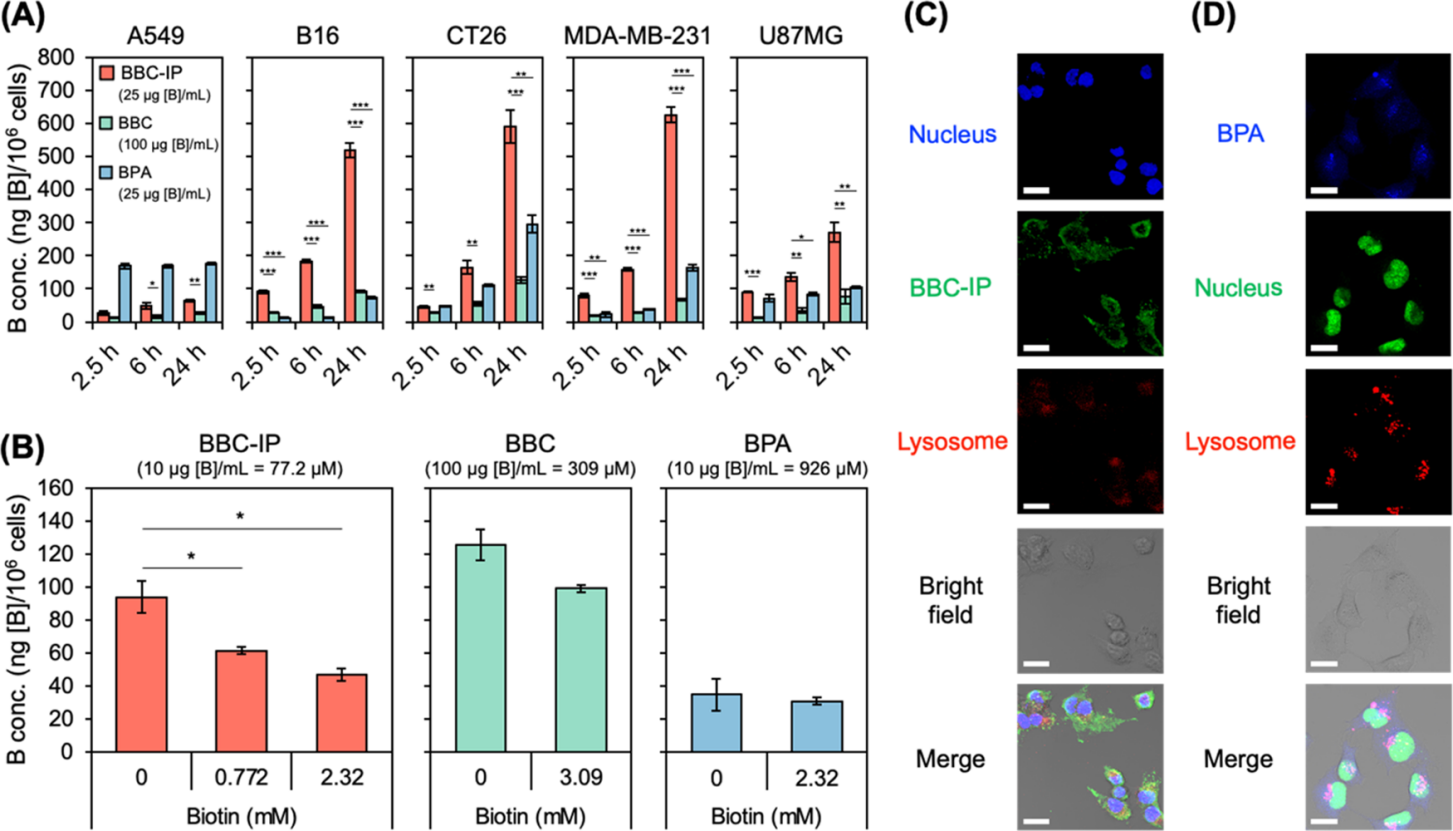
(A) Time-course evaluation of cellular uptake for BBC-IP, BBC, and BPA in A549, B16, CT26, MDA-MB-231, and U87MG cells. Compounds were incubated for 2.5, 6, and 24 h in cells, and boron concentration was measured using inductively coupled plasma optical emission spectroscopy (ICP-OES) after ashing with conc. HNO_3_. Data are shown as the mean ± SEM from a single experiment conducted in triplicate. (B) Biotin concentration-dependent cell uptake study of BBC-IP, BBC and BPA in CT26 cells. Boron concentration was determined after incubation with compounds at various concentrations of biotin for 24 h. (C) Intracellular distribution of BBC-IP in CT26 cells. Cells were incubated with BBC-IP (ca. 25 μg [B]/mL) for 3 h, followed by immunoblotting using an anti-*closo*-dodecaborate antibody and lysosomal staining with Lysotracker. Red: lysosomes stained with LysoTracker Red DND-99; Green: BBC-IP localization; Blue: nuclei stained with DAPI. Scale bars: 25 μm. (D) Intracellular distribution of BPA in CT26 cell lines. Cells were incubated with BPA (ca. 25 μg [B]/mL) for 3 h, followed by fluorescent modification with a boron sensor,^24^ nucleus staining with NucleoSeeing and lysosomal staining with a lysotracker. Red: LysoTracker Red DND-99-stained lysosome; Green: NucleoSeeing reagent-stained nucleus; blue: the localization of BPA. Scale bars: 20 μm. The two-sided Student’s *t*-test was applied to determine statistical significance, defined as *p < 0.05, **p < 0.01, and ***p < 0.001.

We examined the cellular uptake of BBC-IP and BBC in the five cell lines mentioned above using BPA as a control. As shown in Figure 3A, most cell lines showed higher accumulation of BBC-IP than BBC, despite BBC being exposed at a concentration 4 times higher than BBC-IP. In particular, the accumulation of BBC-IP was significantly greater than that of BPA in B16, CT26, MDA-MB-231, and U87MG cells after 24 h of compound treatment. However, A549 cells displayed lower cellular uptake of BBC-IP compared to BPA under similar conditions. According to a literature summarizing biotin receptor expression levels,^11,12^ A549, CT26, and MDA-MB-231 cells exhibit high expression, while B16 cells show low expression, and the expression level in U87MG cells remains unclear. Our findings revealed low uptake of BBC-IP in A549 cells, contrasting with significant accumulation in B16 cells, which have lower biotin receptor expression. This indicates that BBC-IP may be taken up through pathways other than the biotin receptor. Furthermore, we assessed LAT1 expression using Western blot analysis and observed particularly high levels in A549 cells (Figure S3, Supporting Information). After 3 h of BPA exposure, the uptake in A549 cells was notably significant and appeared to have reached equilibrium. This observation suggests a correlation between uptake levels and LAT1 expression, consistent with previous reports indicating that BPA is primarily taken up via LAT1.^3^ We also investigated the cellular uptake under biotin competitive conditions (Figure 3B). The accumulation of BBC-IP and BBC decreased in correlation with the biotin concentration, up to approximately 50 and 20%, respectively. On the other hand, the accumulation of BPA was largely unaffected by the presence of biotin. However, it is important to note that even the observed inhibition of uptake for BBC-IP and BBC was not substantial, suggesting the necessity of considering alternative uptake pathways beyond the targeted biotin receptor. This is particularly evident with BBC, which, due to its simpler structure, exhibits a lower rate of inhibition. Furthermore, Figure 3A demonstrates that there is no correlation between biotin receptor expression levels and the cellular uptake of BBC-IP and BBC across the five cancer cell lines, further emphasizing the likelihood of an independent uptake pathway that operates independently of the targeted biotin receptor. We next examined the subcellular distribution of BBC-IP and BPA in CT26 cells by immunostaining using an anti-*closo*-dodecaborate antibody and a fluorescent boron sensor,^24^ respectively (Figure 3C,D). BBC-IP predominantly localized to the cytosol, including lysosomes, but not to the nucleus. In contrast, BPA was widely distributed throughout the cell, including the nucleus. These results indicate that BBC-IP is a superior boron carrier in terms of intracellular boron concentration, while BPA is superior in terms of subcellular localization in CT26 cells. In parallel with these observations, we investigated the endocytic pathways involved in the uptake of BBC-IP, building on prior studies that suggest receptor-mediated endocytosis for biotin-conjugated molecules.^14^ For example, biotinylated Quantum Dots have been shown to colocalize with clathrin markers,^25^ and biotinylated Gemcitabine accumulates in lysosomes postuptake.^15^ Additionally, the uptake of biotinylated nanoparticles can be inhibited by clathrin-mediated endocytosis inhibitors and macropinocytosis inhibitors,^26^ implying the involvement of a novel endocytic receptor. To further investigate the endocytic mechanism of BBC-IP, we examined its uptake in CT26 cells under three conditions: 37 °C (control), 4 °C (where all endocytic pathways are generally halted), and 37 °C with 100 equiv of sucrose (a known inhibitor of clathrin-mediated endocytosis).^27^ We observed a significant reduction in BBC-IP uptake at 4 °C, consistent with an endocytic mechanism (Figure S4, Supporting Information). However, sucrose treatment at 37 °C caused minimal changes in uptake, indicating that clathrin-mediated endocytosis is not the primary route for BBC-IP. Instead, these results suggest that alternative endocytic pathways, such as caveolar endocytosis or macropinocytosis, similar to the uptake of albumin, may be involved.

### Biodistribution and BNCT Effect

**Figure 4:**
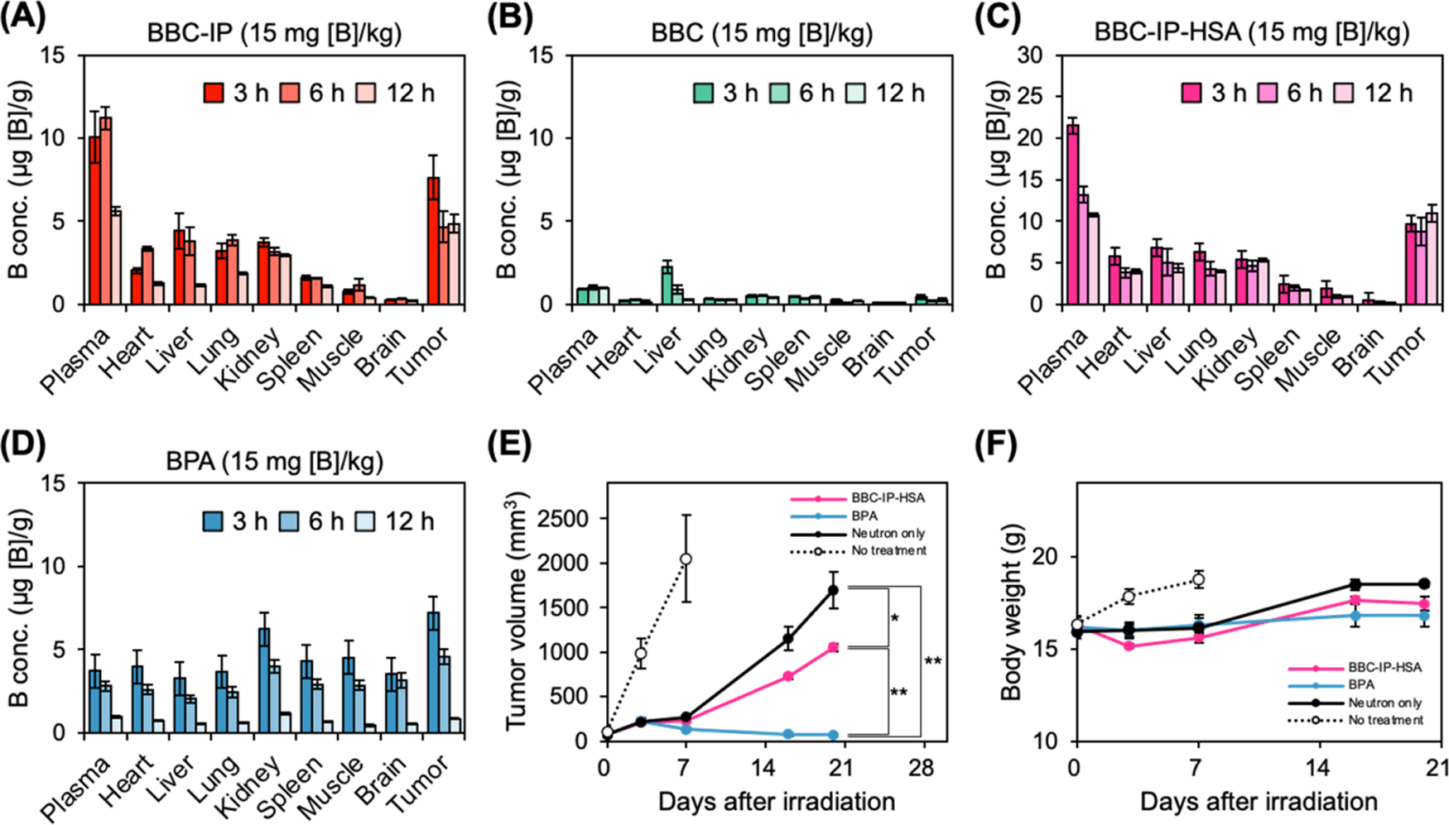
(A–D) Biodistribution of boron in the CT26-bearing mice at 3, 6, and 12 h post- intravenous injection of (A) BBC-IP, (B) BBC, (C) BBC-IP-HSA and (D) BPA at the dose of 15 mg [B]/kg. The data are shown as mean ± SEM (*n* = 3–4). (E, F) BNCT of the CT26 tumor-bearing mice intravenously injected with 15 mg [^10^B]/kg of BBC-IP-HSA and BPA. (E) The tumor volume curves after thermal neutron irradiation (also see Figure S5, Supporting Information). (F) Mice body weight corresponding to the treatments in panel (E). Results are shown as the mean ± SEM (*n* = 5 for the groups with BBC-IP-HSA, BPA and no treatment, *n* = 4 for the groups with neutron only). The two-sided Student’s *t*-test was applied to determine statistical significance, defined as *p < 0.05, **p < 0.0001.

The biodistribution study was conducted in CT26 tumor-bearing mice, and boron concentrations in various organs were measured by inductively coupled plasma optical emission spectroscopy (ICP-OES). Mice were intravenously administered each boron compound shown below at a dose of 15 mg [B]/kg, and biodistribution was evaluated at 3, 6, and 12 h postadministration. BBC-IP exhibited superior blood retention and tumor accumulation compared to BBC (Figure 4A,4B). These results suggest that this enhanced performance is attributed to the binding of BBC-IP to albumin in the body via an albumin ligand within the molecule, thus contributing to the long-term blood retention and the EPR effect. We next evaluated the biodistribution of albumin complexes (BBC-IP-HSA) formed by the interaction of BBC-IP with albumin *in vitro* (Figure 4C). The tumor boron concentrations at 3, 6, and 12 h postadministration of BBC-IP-HSA were 9.7, 8.9, and 10.9 μg [B]/g, respectively, indicating improved tumor accumulation compared to BBC-IP alone. Furthermore, the maximum tumor boron concentration in mice treated with BPA was 7.2 μg [B]/g at 3 h postinjection (Figure 4D), suggesting that BBC-IP-HSA achieved higher levels of boron accumulation than BPA.

We subsequently assessed the BNCT efficacy of BBC-IP-HSA in CT26 tumor-bearing mice. The mice were intravenously injected with boron-10-enriched BBC-IP-HSA, BBC-IP, and BPA at a dose of 15 mg [^10^B]/kg. After 3 h, the tumors were then subjected to thermal neutron irradiation at a dose of 3.2–3.3 × 10^12^ neutrons/cm^2^. Tumor volume changes in mice were monitored over a 20-day period. The mice treated with BBC-IP-HSA and BPA showed significantly greater tumor suppression compared to the neutron-only group 20 days after neutron irradiation (Figure 4E). Furthermore, mice treated with BBC-IP-HSA and BBC-IP exhibited similar tumor growth behavior (Figure S5, Supporting Information). Contrary to expectations from the biodistribution study, BPA showed significantly higher antitumor efficacy than BBC-IP-HSA. This observation suggested one possibility that the observed antitumor effects can be attributed to differences in intracellular localization between BBC-IP and BPA. Previous studies have investigated the relationship between the intracellular localization of boron compounds and the energy delivered to the nucleus by the particle beam generated during BNCT, suggesting that proximity of boron compounds to the nucleus correlates with higher BNCT efficiency.^28^ As demonstrated in Figure 3C,D, BBC-IP localizes predominantly in the cytoplasm, including lysosomes, while BPA exhibits widespread distribution throughout the cell, including the nucleus. These results indicate that differences in intracellular localization play a more significant role in BNCT efficacy than the quantity of boron accumulated in tumors. Furthermore, BNCT using BBC-IP-HSA did not cause any notable side effects, as evidenced by no significant differences in mouse body weights across all groups (Figure 4F). These observations indicate that although BNCT with BBC-IP-HSA is effective against CT26 tumors, its efficacy is still inferior to that of BPA.

## Conclusions

Our study highlights the potential of the novel small molecule boron carrier BBC-IP, targeting biotin receptors overexpressed in a variety of cancer cells, as a promising candidate for BNCT. BBC-IP demonstrated remarkable attributes such as high water solubility, minimal cytotoxicity, and superior cellular uptake compared to the approved BNCT agent BPA in both human and mouse cancer cells. Biodistribution studies revealed that BBC-IP significantly enhanced tumor accumulation, particularly in mouse colon tumors, surpassing the accumulation levels of BPA. Despite its enhanced tumor accumulation, BBC-IP did not exhibit superior BNCT efficacy compared to BPA. These results suggest that while improved tumor targeting and accumulation are essential, the intratumoral localization is also crucial for the efficacy of novel BNCT agents. Therefore, further optimization of the intracellular localization of BBC-IP could enhance BNCT efficacy and provide more effective options for future cancer therapy.

## Experimental Procedures

### Chemicals

The chemicals were obtained from BLD Pharmatech (Shanghai, China), FUJIFILM Wako Pure Chemical (Osaka, Japan), Nacalai Tesque (Kyoto, Japan), Sigma-Aldrich (Burlington, MA), and Tokyo Chemical Industries (Tokyo, Japan). All reagents were of the highest available grade and used as received without additional purification. Na_2_[^10^B_12_H_12_] was kindly supplied by Stella Pharma Co., Ltd. (Osaka, Japan). (Et_3_NH)_2_[B_12_H_12_] was obtained from Katchem Ltd. (Praha, Czech Republic). TLC analysis was performed on silica gel 60 F254 glass plates (Merck) under UV light (254 nm). Column chromatography used CHROMATOREX PSQ 60B silica gel (50–200 μm, Fuji Silysia). NMR spectra were obtained using a Bruker AVANCE III-500 spectrometer (500 MHz for ^1^H, 125 MHz for ^13^C, 160 MHz for ^11^B) with DMSO-*d*_6_ or D_2_O as solvents. Chemical shifts are reported in ppm, relative to DMSO-*d*_6_ (2.50 ppm for ^1^H, 39.52 ppm for ^13^C) or D_2_O (4.79 ppm for ^1^H). ^11^B NMR shifts were externally referenced to BF_3_·Et_2_O (0.00 ppm). Signal multiplicities are abbreviated as s (singlet), d (doublet), dd (doublet of doublets), t (triplet), quin (quintet), m (multiplet), and J (coupling constants in Hz). IR spectra were recorded using a JASCO FT/IR-4200. HRMS data were collected with a Bruker ESI-TOF-MS (micro TOF II). HPLC analysis was performed using a JASCO PU-4086-Binary Semipreparative Pump, UV-4075 UV/vis Detector, and LC-NetII/ADC Interface Box to confirm compound purity.

### Chemical Synthesis of BBC-IP

To a stirred mixture of biotin (413 mg, 1.69 mmol, 1.2 equiv), amine **1**(10a) (1.87 g, 1.41 mmol, 1.0 equiv) and 1-hydroxybenzotriazole monohydrate (HOBt·H_2_O; 378 mg, 1.97 mmol, 1.4 equiv) in *N*,*N*-dimethylformamide (DMF; 20 mL), 1-(3-(dimethylamino)propyl)-3-ethylcarbodiimide hydrochloride (EDCI·HCl; 268 mg, 1.97 mmol, 1.4 equiv) in DMF (5 mL) and *N*,*N*-diisopropylethylamine (DIEA; 343 μL, 1.97 mmol, 1.4 equiv) was added dropwise at 0 °C under an argon atmosphere. After stirring for 40 h at room temperature, the reaction mixture was concentrated *in vacuo*, yielding a crude product of BBC-IP·2TBA^+^ as a white solid. The product was used in the next step without additional purification and dissolved in ethanol (EtOH; 10 mL). Tetramethylammonium (TMA) chloride (1.63 g, 14.8 mmol, 10 equiv) dissolved in EtOH (10 mL) was gradually added to the solution under stirring. The mixture was allowed to stir at room temperature for 1 h, after which a white precipitate formed. This precipitate was collected via filtration, rinsed with EtOH and CH_2_Cl_2_, and dried *in vacuo*, yielding BBC-IP·2TMA^+^ as a white solid. The solid was subsequently dissolved in a 1:1 acetonitrile (MeCN) and water mixture (60 mL) and stirred with Amberlite-Na^+^ resin (21.7 g) for 18 h at room temperature. After filtration and removal of solvents under reduced pressure, the final product, BBC-IP, was obtained as a light yellowish solid (1.40 g, 1.26 mmol, 89% overall yield across three steps). Mp 262 °C. ^1^H NMR (500 MHz, DMSO-*d*): δ 8.00 (t, *J* = 5.5 Hz, 1H), 7.96 (d, *J* = 8.0 Hz, 1H), 7.84 (t, *J* = 5.6 Hz, 1H), 7.73 (t, *J* = 5.6 Hz, 1H), 7.61 (d, *J* = 8.2 Hz, 2H), 7.00 (d, *J* = 8.2 Hz, 2H), 6.41 (s, 1H), 6.34 (s, 1H), 4.37–4.25 (m, 1H), 4.18–4.11 (m, 1H), 4.11–4.03 (m, 1H), 3.43–3.36 (m, 4H), 3.24–3.05 (m, 4H), 3.05–2.93 (m, 6H), 2.82 (dd, *J* = 12.5 Hz, 5.1 Hz, 1H), 2.61–2.54 (m, 1H), 2.50–2.46 (m, 2 H), 2.43–2.21 (m, 4 H), 2.10–1.98 (m, 4 H), 1.75 (quin, *J* = 7.6 Hz, 2H), 1.70–1.15 (m, 21H); ^11^B NMR (160 MHz, DMSO-*d*): δ 6.56, −16.9, −18.3, −23.2; ^13^C NMR (125 MHz, DMSO-*d*): δ 171.9, 171.8, 171.8, 171.6, 171.6, 162.8, 141.6, 137.0, 130.9, 91.3, 71.9, 69.0, 67.3, 61.1, 59.3, 55.4, 52.7, 38.9, 38.5, 38.4, 38.3, 35.3, 34.7, 34.1, 31.4, 30.8, 30.7, 29.1, 28.9, 28.9, 28.3, 28.1, 26.9, 26.1, 26.0, 25.3, 23.0; HRMS (ESI-TOF): calcd for [C_40_H_74_B_12_IN_7_NaO_8_S]^−^, 1092.5456: found 1092.5456; FT-IR (ATR): 3566, 3366, 2928, 2860, 2480, 1643, 1537, 1458, 1254, 1159, 1113, 1069, 1026, 932, 864, 760, 723 cm^–1^, Analytical HPLC: purity = 98.7%, *t*_R_ = 29.83 min (measurement wavelength, 254 nm; flow rate, 1.0 mL/min; eluent A, phosphate-buffered saline; eluent B, acetonitrile; gradient, 0–30 min, 0–40% B, 30–60 min, 40% B; column, JASCO, Finepak SIL C18S 5 μM, 4.6 mm × 150 mm).

### Chemical Synthesis of BBC

To a stirred mixture of biotin (201 mg, 0.823 mmol, 1.2 equiv), amine **2**(6b) (499 mg, 0.684 mmol, 1.0 equiv) and HOBt·H_2_O (130 mg, 0.965 mmol, 1.4 equiv) in DMF (16 mL), EDCI·HCl (184 mg, 0.962 mmol, 1.4 equiv) in DMF (10 mL) and DIEA (155 μL, 0.890 mmol, 1.3 equiv) was added dropwise at room temperature under an argon atmosphere. After stirring for 14 h at room temperature, the reaction mixture was concentrated *in vacuo*, yielding a residue that was purified by silica gel column chromatography (CH_2_Cl_2_/methanol (MeOH) = 9:1) to give BBC·2TBA^+^ as a yellow oil. The obtained yellow oil was dissolved in MeOH (8 mL). Tetramethylammonium (TMA) chloride (999 mg, 9.12 mmol, 10 equiv) dissolved in MeOH (6 mL) was gradually added to the solution under stirring. The mixture was allowed to stir at room temperature for 30 min, after which a white precipitate formed. This precipitate was collected by filtration, rinsed with EtOH, and dried *in vacuo*, yielding BBC·2TMA^+^ as a white solid. The solid was subsequently dissolved in water (H_2_O; 20 mL) and stirred with Amberlite-Na^+^ resin (11.3 g) for 16 h at room temperature. After filtration, removal of solvents under reduced pressure, and lyophilization, BBC was obtained as a light yellowish solid (116 mg, 0.225 mmol, 33% yield across three steps). Mp 257 °C. ^1^H NMR (500 MHz, D_2_O): δ 4.65 (dd, *J* = 8.0 Hz, 5.0 Hz, 1H), 4.50 (dd, *J* = 7.5 Hz, 4.5 Hz, 1H), 3.71–3.62 (m, 6H), 3.43–3.38 (m, 3H), 3.07 (dd, *J* = 8.0 Hz, 5.0 Hz, 1H), 2.80 (d, *J* = 13.1 Hz, 1H), 2.37–2.26 (m, 2H), 1.81–1.56 (m, 4H), 1.74 (quin, *J* = 7.7 Hz, 2H); ^11^B NMR (160 MHz, D_2_O): δ 6.56, – 16.4, – 18.3, – 23.5; ^13^C NMR (125 MHz, D_2_O): δ 177.0, 165.4, 70.9, 68.6, 67.6, 62.1, 60.3, 55.3, 39.7, 39.1, 35.5, 27.8, 27.7, 25.2; HRMS (ESI-TOF): calcd for [C_14_H_35_B_12_N_3_NaO_4_S]^−^, 494.3440: found 494.3444; FT-IR (ATR): 3561, 3387, 2930, 2870, 2473, 1678, 1628, 1545, 1460, 1429, 1356, 1331, 1265, 1153, 1109, 1059, 1032, 922, 870, 760, 721 cm^–1^, Analytical HPLC: purity = 97.8%, *t*_R_ = 8.67 min (measurement wavelength, 254 nm; flow rate, 1.0 mL/min; eluent A, phosphate-buffered saline; eluent B, acetonitrile; gradient, 0–15 min, 0–20%; column, JASCO, Finepak SIL C18S 5 μM, 4.6 mm × 150 mm).

### Binding Affinity of Boron Compounds to Human Serum Albumin (HSA) Determined by Enzyme-Linked Immunosorbent Assay (ELISA)

A protein-binding 96-well plate (Griner Bio-one, Inc., Germany) was coated with 100 μM HSA dissolved in PBS and incubated at 37 °C for 12 h. After washing 3 times with PBS, the plate was blocked using ELISA blocking reagent (Immnoblock, KAC Co., Ltd., Japan) at room temperature for 1.5 h. Subsequently, PBS (standard) or boron compound solutions (concentration range: 316 nM to 31.6 mM) were added, and the plate was incubated at room temperature for another 1.5 h. For detection, the plate was incubated with rabbit anti-MID IgG as the primary antibody for 1.5 h at room temperature, followed by goat antirabbit IgG (H&L) conjugated with Alexa Fluor 488 (#ab150077, Abcam, U.K.) as the secondary antibody for 1.5 h at room temperature. The rabbit anti-MID IgG was custom-made and supplied by Scrum Co., Ltd. (Japan). Fluorescence intensity was measured using a microplate reader (infinite F200, Tecan Japan Co., Ltd., Japan) with excitation/emission wavelengths of 485/535 nm. The data were analyzed with a four-parameter logistic equation to produce a sigmoidal curve using ImageJ software (National Institutes of Health).

### Dynamic Light Scattering (DLS) and ζ-Potential Analysis

BBC-IP solutions at concentrations of 0, 1500, 3000, and 6000 μg [B]/mL were prepared in 500 μL of PBS and added to 500 μL of HSA (30.8 mg) also in PBS. The final concentration of HSA was 0.463 mM, while the final concentrations of BBC-IP were 0, 750, 1500, and 3000 μg [B]/mL, corresponding to 0, 12.5, 25, and 50 equiv of HSA, respectively. After incubation at 37 °C for 24 h, 1 mL of each sample was transferred to Disposable Folded Capillary cell (DTS1070, Malvern Panalytical, Ltd., U.K.). These tubes were then placed in the sample compartment of the Zetasizer Nano Series (Malvern Panalytical), and the average particle size, distribution width, and ζ-potential of the sample BBC-IP were measured. The particle size and distribution width were measured using a scattering angle of 173°, a solvent refractive index of 1.33, a solvent viscosity of 0.89, and 3 number of measurements. The ζ-potential was determined using the electrophoretic mobility method at 25 °C with 3 number of measurements. Data analysis was conducted using the software provided by Malvern Panalytical, and the results were reported as the mean ± standard deviation of the measurements.

### Field Emission Scanning Electron Microscopy (FE-SEM)

The microgrid was treated for 40 s using a hydrophilization device (HDT-400, JEOL). One drop of the BBC-IP-HSA solution (1.23 mM BBC-IP and 0.082 mM HSA) was then placed onto the treated microgrid. After 60 s, the excess solution was removed with filter paper. The sample was dried overnight in a DRY CABI. It was then observed using an FE-SEM (S-5500, Hitachi High-Technologies) with an acceleration voltage of 1 kV. No metal coating was applied.

### Cell Culture and Cytotoxicity

Human lung carcinoma (A549), mouse melanoma (B16), mouse colon carcinoma (CT26), human breast adenocarcinoma (MDA-MB-231), and human glioblastoma (U87MG) cell lines were maintained in Roswell Park Memorial Institute (RPMI)-1640 medium (Fujifilm Wako Pure Chemicals Co., Ltd., Japan) supplemented with 10% heat-inactivated fetal bovine serum (Thermo Fisher Scientific, Inc.) and 1% penicillin/streptomycin (final concentrations: 100 units/mL penicillin G and 10 μg/mL streptomycin; Thermo Fisher Scientific, Inc.). The cells were cultured at 37 °C in a humidified incubator with 5% CO_2_. For experiments, A549, B16, CT26, MDA-MB-231, and U87MG cells were seeded into 96-well plates at a density of 1000 cells per well. After 24 h, the cells were treated with BBC-IP, BBC, or BPA at final concentrations ranging from 1 μM to 1 mM for 72 h. Following treatment, 0.5 mg/mL 3-(4,5-dimethylthiazol-2-yl)-2,5-diphenyltetrazolium bromide (MTT; Tokyo Chemical Industry Co., Ltd., Japan) was added to each well, and the plates were incubated for 4 h at 37 °C. After incubation, the medium was carefully removed, and the resulting MTT formazan product was dissolved in 100 μL of dimethyl sulfoxide (DMSO). The absorbance of the solution was measured at 590 nm using a microplate reader. The half-maximal inhibitory concentration (IC_50_) for each compound was calculated from semilogarithmic dose–response curves.

### Time-Course Analysis of Cellular Uptake

A549, B16, CT26, MDA-MB-231, and U87MG cells were seeded in 60 mm dishes at 1.0 × 10^6^ cells/dish. After incubation for 24 h, the cells were treated with BBC-IP, BPA (ca. 25 μg [B]/mL), and BBC (ca. 100 μg [B]/mL) for 2.5, 6, and 24 h. The cells were then collected using a cell scraper. The cell count for each sample was performed, and the cells were washed in conc. HNO_3_ (Kanto Chemical Co., Inc., Japan) at 70 °C for 1 h. The resulting solutions were diluted with Milli-Q water and filtered through a 0.5 μm polypropylene filter. Boron concentrations were measured by inductively coupled plasma optical emission spectroscopy (ICP-OES, iCAP 7400 Duo, Thermo Fisher Scientific Inc.).

### Competition Assay

CT26 cells were seeded in 100 mm dishes at 1.0 × 10^6^ cells/dish. After a 24 h-incubation, the cells were treated with BBC-IP (ca. 10 μg [B]/mL = 77.2 μM), BPA (ca. 10 μg [B]/mL = 926 μM), and BBC (ca. 100 μg [B]/mL = 309 μM). Subsequently, biotin (772 μM to 3.09 mM) was added. The cells were incubated for another 24 h, harvested using a cell scraper, and counted. The collected cells were ashed in conc. HNO_3_ at 70 °C for 1 h. The resulting solutions were diluted with Milli-Q water and filtered through a 0.5 μm polypropylene filter and their boron concentrations were identified by ICP-OES.

### Intracellular Localization of BBC-IP with Lysotracker by Immunostaining

CT26 cells were seeded on cover glasses in a 6-well plate at 1.0 × 10^5^ cells/well and incubated for 12 h. After the medium was replaced with fresh medium containing BBC-IP (ca. 25 μg [B]/mL), the cells were incubated for 3 h and then washed with PBS. Subsequently, the cells were incubated with fresh medium containing 100 nM Lysotracker Red DND-99 (Thermo Fisher Scientific, Inc.) at 37 °C for 30 min. The cells were then washed 3 times with PBS, fixed with 4% paraformaldehyde (PFA, Fujifilm Wako Pure Chemicals Co., Ltd., Japan) for 10 min, and permeabilized with 0.1% Triton X-100 (Nacalai Tesque, Inc., Japan) for 5 min. After blocking with Immnoblock for 30 min, the cells were sequentially incubated with rabbit anti-MID IgG and goat Alexa Fluor 488-conjugated antirabbit IgG (H&L) at room temperature for 1.5 h each. The intracellular nuclei were stained with Hoechst 33342 (Dojindo Laboratories, Japan). Fluorescence signals were recorded using a confocal laser microscope (LMS780 spectral confocal system, Zeiss Co., Ltd., Germany).

### Intracellular Localization of BPA with Boron Sensor and Lysotracker

CT26 cells were seeded on cover glasses in a 6-well plate at 2.0 × 10^5^ cells/well and incubated for 12 h. After the medium was replaced with fresh medium containing BPA (ca. 25 μg [B]/mL), the cells were further incubated for 3 h and washed with PBS. Subsequently, the cells were incubated with fresh medium containing 1 mM boron sensor, 100 nM Lysotracker Red DND-99, and 1 μM NucleoSeeing reagent (Funakoshi Co., Ltd., Japan) at 37 °C for 30 min. The cells were washed 3 times with PBS and fixed with 4% paraformaldehyde for 10 min. Fluorescence signals were recorded using a confocal laser microscope.

### Preparation of BBC-IP-HSA

To a solution of BBC-IP (138.1 mg, 124 μmol, 25 equiv) in PBS (4.0 mL) was added HSA (327.8 mg, 4.93 μmol, 1 equiv). After stirred at 37 °C for 1 day, the mixture was filtered through a 0.5 μm polypropylene filter, adjusted to a concentration of 1500 μg [B]/mL using ICP-OES. The resulting BBC-IP solution was used for *in vivo* experiments. All animal experiments were conducted in accordance with the Guide for the Care and Use of Laboratory Animals and approved by the Animal Use Review Board and Ethical Committee of Institute of Science Tokyo and Institute for Integrated Radiation and Nuclear Science, Kyoto University.

### Biodistribution of Boron Agents in CT26 Xenograft Models

CT26 tumor-bearing mice (BALB/cSlc, female, 5–6 weeks old, 14–20 g, Sankyo Labo Service Co., Ltd., Japan) were prepared by subcutaneous injection of a suspension of CT26 cells (1.0 × 10^6^ cells/mouse) into the right thigh. When the tumor diameter reached 5 to 7 mm, the mice were intravenously injected with 200 μL of PBS solutions containing BBC-IP-HSA, BBC-IP, BBC, or BPA (15 mg [B]/kg) via the tail vein. At 3, 6, and 12 h postinjection, the mice were lightly anesthetized, and blood samples were collected by cardiac puncture. The mice were sacrificed by cervical dislocation, and their heart, liver, lungs, kidneys, spleen, muscles, brain, and tumor were carefully excised. Each organ was rinsed with 0.9% NaCl solution, weighed, and digested in 1 mL of concentrated HNO_3_ at 90 °C for 1 h. The digested samples were then diluted with distilled water, filtered using a hydrophobic membrane, and analyzed for boron concentrations using ICP-OES.

### BNCT Efficacy of Boron Compounds in CT26 Xenograft Models

CT26 tumor-bearing mice were prepared by subcutaneously injecting a suspension of CT26 cells (8.0 × 10^5^ cells/mouse) into the right thigh. The mice were maintained on a standard diet with free access to water under a 12 h light/dark cycle in an ambient environment. When the tumor diameter reached 5 to 7 mm, the mice were intravenously injected with 200 μL of PBS containing BBC-IP-HSA, BBC-IP, or BPA (15 mg [^10^B]/kg) via the tail vein. At 3 h postadministration, the mice were placed in an acrylic holder fixed on a 5 mm-thick thermoplastic plate, and the right thighs were irradiated with neutrons using a nuclear reactor (3.2–3.3 × 10^12^ neutrons/cm^2^). The BNCT effects were assessed based on tumor volume changes. Tumor volume was calculated using the formula 0.5 × (*A* × *B*^2^), where *A* and *B* represent the longest and shortest tumor diameters in millimeters, respectively. Mice were euthanized when their tumor volumes exceeded 2000 mm^3^.

## Supporting Information Available

Binding affinity of BBC-IP and PBC-IP toward HSA (Figure S1); UV–vis spectra of BBC-IP and BBC under stability testing conditions at 37 °C over a period of 6 days (Figure S2); evaluation of LAT1 expression levels in each cancer cell lines (Figure S3); uptake of BBC-IP in CT26 cells under different conditions: 37 °C (control), 4 °C (endocytosis-inhibited), and 37 °C with 100 equiv of sucrose (inhibitor of clathrin-mediated endocytosis) (Figure S4); tumor growth curves of the individual mice (Figure S5), and NMR spectra and HPLC chromatogram of BBC-IP and BBC (PDF)

## Abbreviations

BBC: biotin-*closo*-dodecaborate conjugate
BBC-IP: biotinyl-*closo*-dodecaborate conjugate with an iodophenyl moiety
BNCT: boron neutron capture therapy
BPA: 4-borono-l-phenylalanine
BRs: biotin receptors
DLS: dynamic light scattering
DMSO: dimethyl sulfoxide
ELISA: enzyme-linked immunosorbent assay
EPR: enhanced permeability and retention
FA: fatty acid
FE-SEM: field emission scanning electron microscopy
HSA: human serum albumin
ICP-OES: inductively coupled plasma optical emission spectroscopy
IC_50_: median inhibitory concentration
*K*_b_: binding constant
*K*_d_: dissociation constant
LAT1: L-type amino acid transporter 1
MID: maleimide-functionalized *closo*-dodecaborate
MRI: magnetic resonance imaging
MTT: 3-(4,5-dimethyl-2-thiazolyl)-2,5-diphenyltetrazolium bromide
PBC-IP: pteroyl-*closo*-dodecaborate conjugated with a 4-(*p*-iodophenyl)butyric acid moiety
PBS: phosphate buffer solution
SMVT: sodium-dependent multivitamin transporter
