# Supplementary material for: A Water-Soluble Small Molecule Boron Carrier Targeting Biotin Receptors for Neutron Capture Therapy
Source: ACS Omega. 2024 Dec 18;9(52):51631–40. doi: 10.1021/acsomega.4c09388 (PMC11696430; doi:10.1021/acsomega.4c09388)
Supplement: Supplementary file 1 — ao4c09388_si_001.pdf [file ao4c09388_si_001.pdf]

Supporting Information

A water-soluble small molecule boron carrier targeting biotin receptors for neutron capture therapy

Kai Nishimura,[1,2] *Shota Tanaka,*[2] *Kazuki Miura,*[1,2] *Satoshi Okada,*[1,2] *Minoru Suzuki,*[3] *and Hiroyuki Nakamura**[,1,2]

[1]School of Life Science and Technology, Institute of Science Tokyo, 4259 Nagatsuta-cho Midori-ku, Yokohama, 226-8501, Japan

[2]Laboratory for Chemistry and Life Science, Institute of Integrated Research, Institute of Science Tokyo, 4259 Nagatsuta-cho Midori-ku, Yokohama, 226-8501, Japan

[3]Institute for Integrated Radiation and Nuclear Science, Kyoto University, 2-1010, Asashiro-Nishi, Kumatori-cho, Sennan-gun, Osaka 590-0494, Japan

*Corresponding Author:
Laboratory for Chemistry and Life Science, Institute of Integrated Research, Institute of Science Tokyo, Yokohama, Kanagawa 226-8501, Japan; School of Life Science and Technology, Institute of Science Tokyo, Yokohama, Kanagawa 226-8501, Japan
E-mail: hiro@res.titech.ac.jp (H. Nakamura)

Table of contents

1. Supplementary figures S2

2. ^{1}H, ^{11}B, and ^{13}C NMR spectra S7

3. HPLC chromatograms S10

1. Supplementary figures

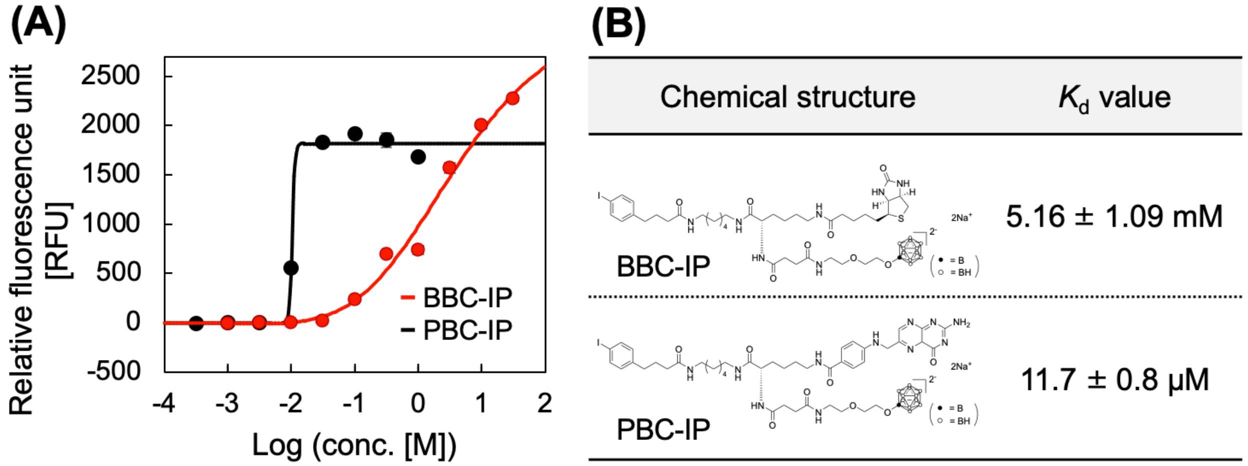

Figure S1. Binding affinity of BBC-IP and PBC-IP toward HSA. (A) Semi-Log dose response curves for the binding affinity of BBC-IP and PBC-IP. (B) Calculated dissociation constant (K_d value) of BBC-IP and PBC-IP. Data are expressed as means ± SEM from a single experiment conducted in triplicate.

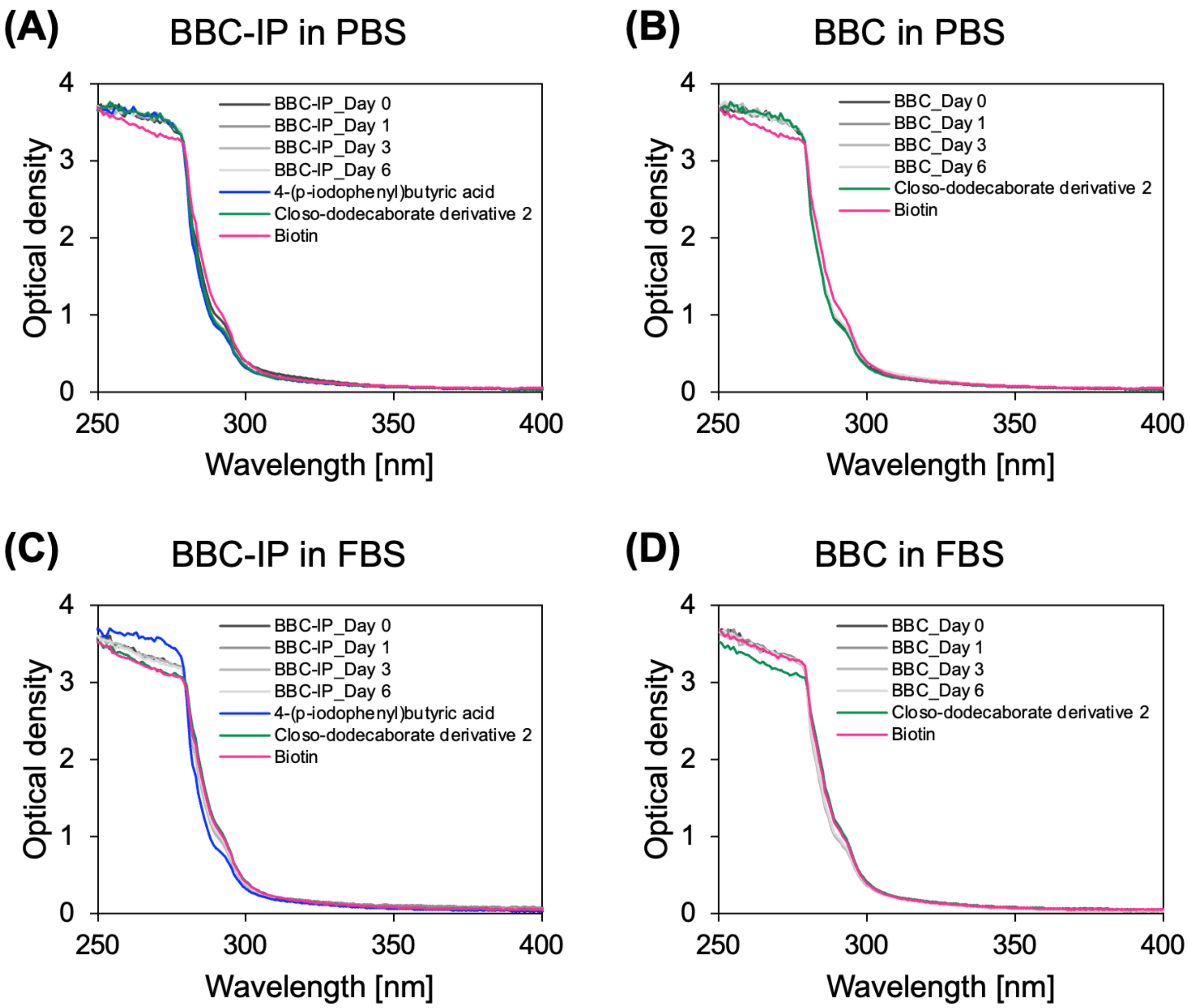

Figure S2. UV-Vis spectra of BBC-IP and BBC under stability testing conditions at 37 °C over a period of 6 days. Measurements were conducted in (A) PBS with BBC-IP, (B) PBS with BBC, (C) FBS with BBC-IP, and (D) FBS with BBC. Each spectrum was recorded daily. Spectra of potential decomposition products, including iodophenyl butyric acid, *closo*-dodecaborate deribative **2**, and biotin, are also shown for comparison.

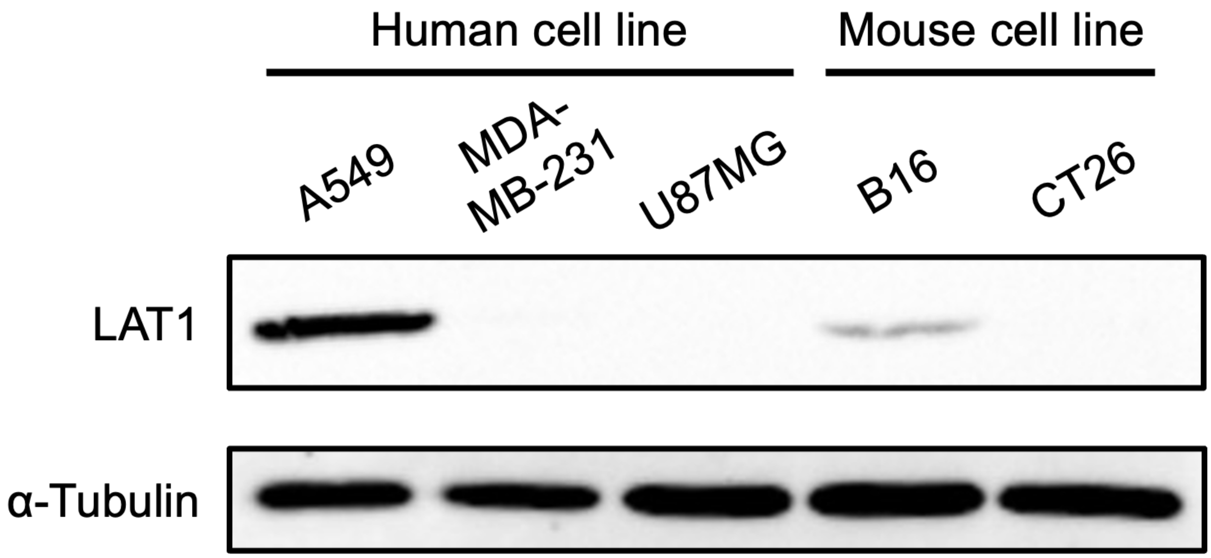

Figure. S3. Evaluation of LAT1 expression levels in each cancer cell lines. Each cancer cells were lysed and then the obtained cell lysates were separated by SDS-PAGE. Immunoblotting was performed using anti-LAT1 and anti-α-tubulin antibodies. α-Tubulin was used as an internal control.

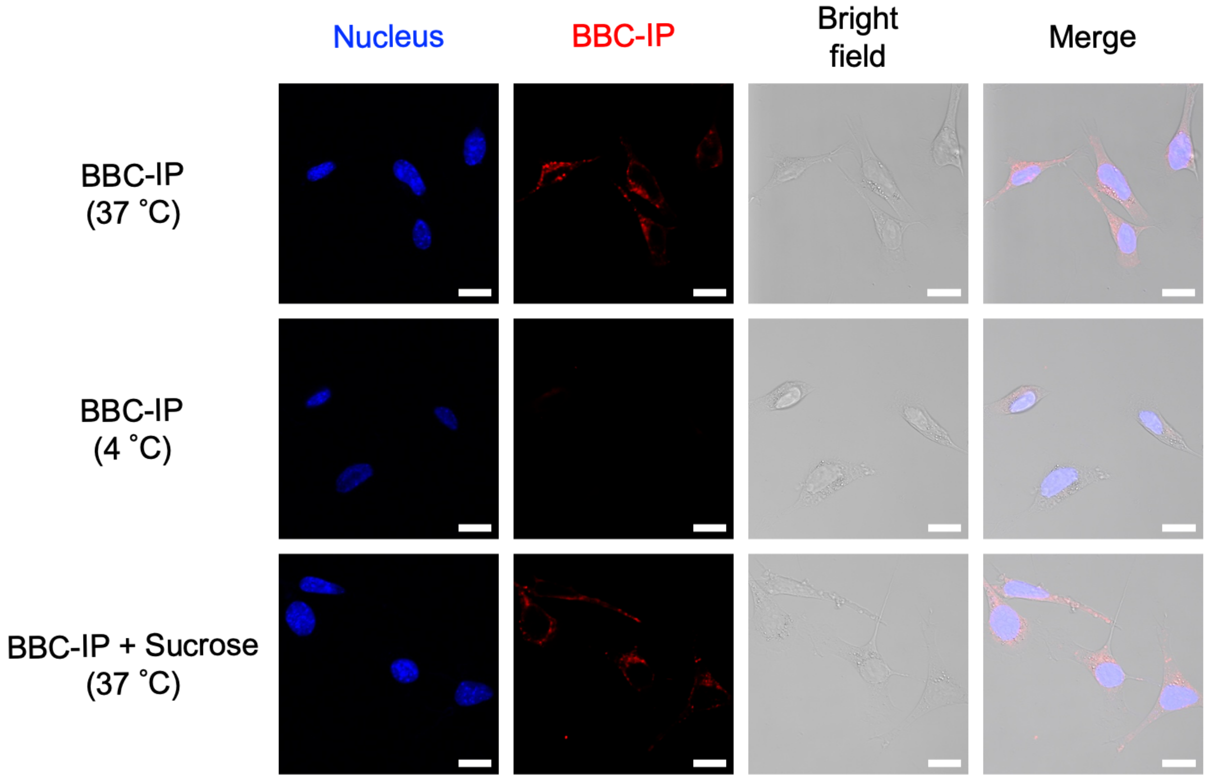

Figure S4. Uptake of BBC-IP in CT26 cells under different conditions: 37°C (control), 4°C (endocytosis-inhibited), and 37°C with 100 equivalents of sucrose (inhibitor of clathrin-mediated endocytosis). Cells were incubated with BBC-IP (ca. 25 μg [B]/mL) for 3 h in each condition, followed by immunostaining with an anti-*closo*-dodecaborate antibody. Red: the localization of BBC-IP; blue: DAPI-stained nuclei. Scale bar: 20 μm.

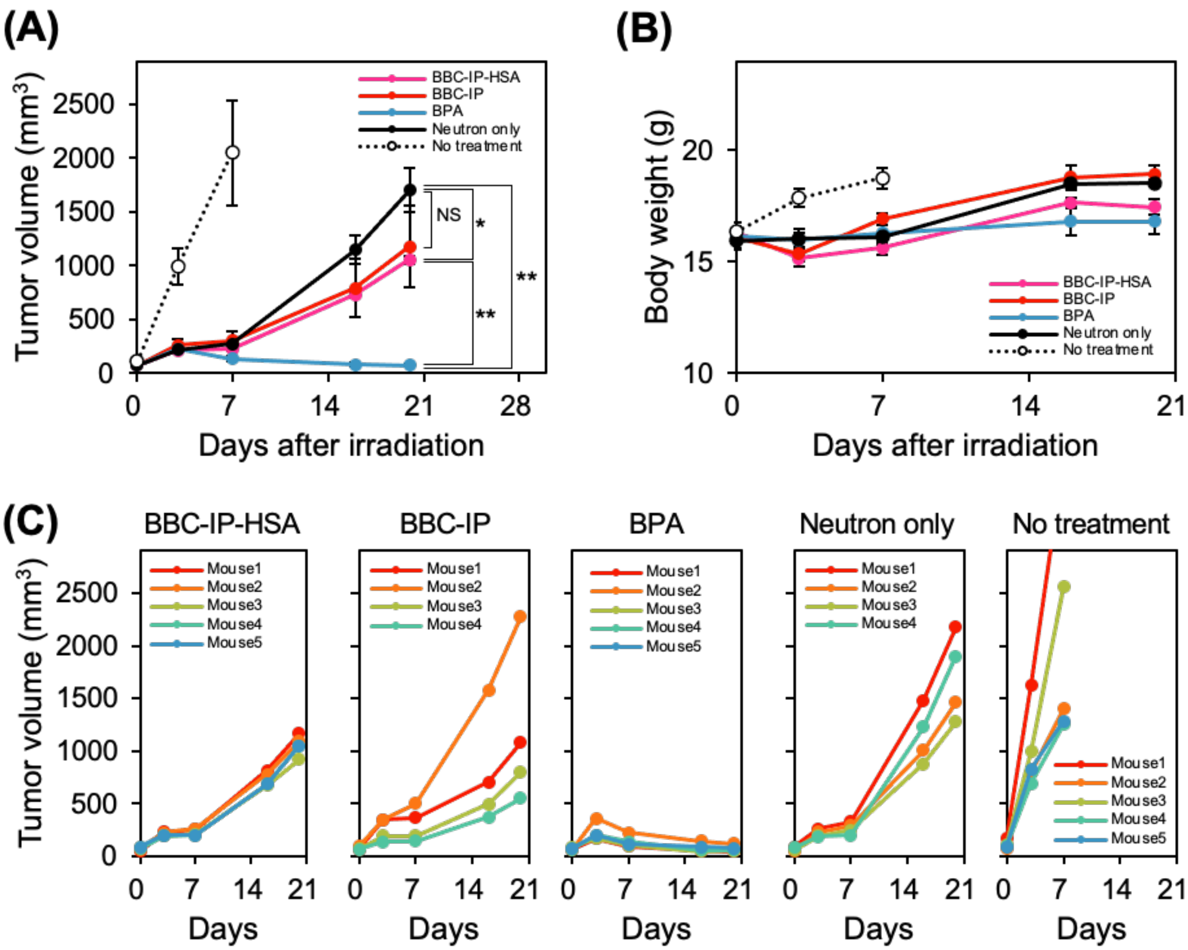

Figure S5. BNCT of the CT26 tumor-bearing mice intravenously injected with 15 mg[^{10}B]/kg of BBC-IP-HSA, BBC-IP, and BPA. (A) The tumor volume curves after thermal neutron irradiation. (B) Body weight of the same mice treated in panel A. (C) The tumor volume curves of the individual mice after irradiation with thermal neutron. The data are shown as mean ± SEM (n = 5 for the groups with BBC-IP-HSA, BPA and no treatment, n = 4 for the groups with BBC-IP and neutron only). Significance was determined as: *p < 0.05, **p < 0.0001 using the two-sided Student's t-test.

2. NMR spectra

BBC-IP

^{1}H NMR (500 MHz, DMSO-d_6)

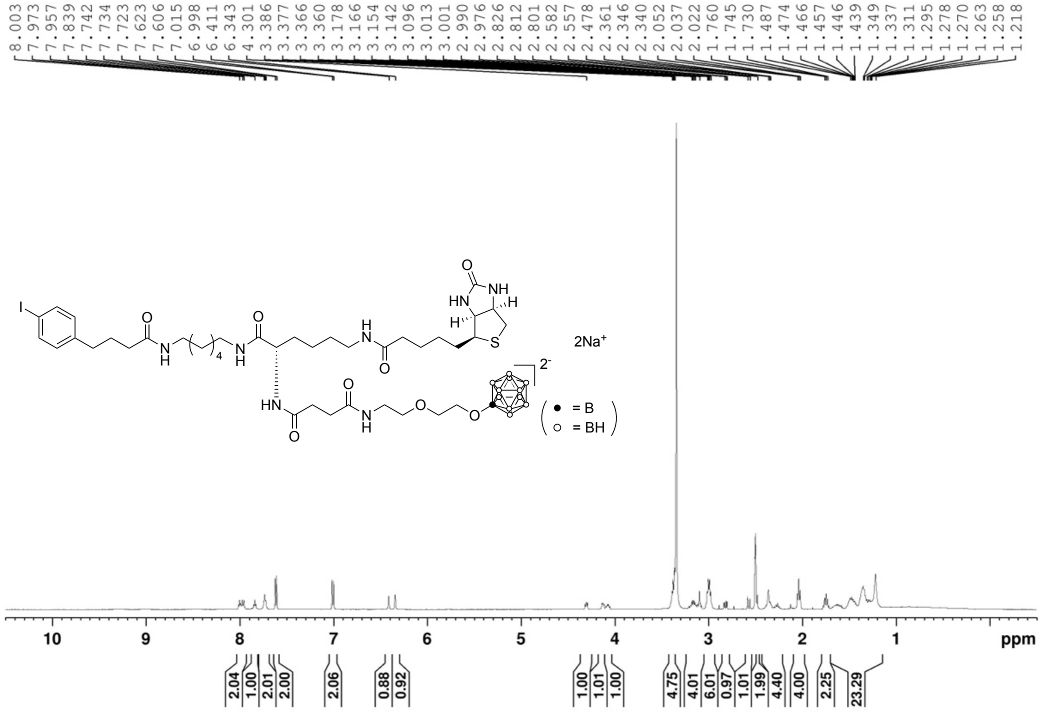

^{11}B NMR (160 MHz, DMSO-d_6)

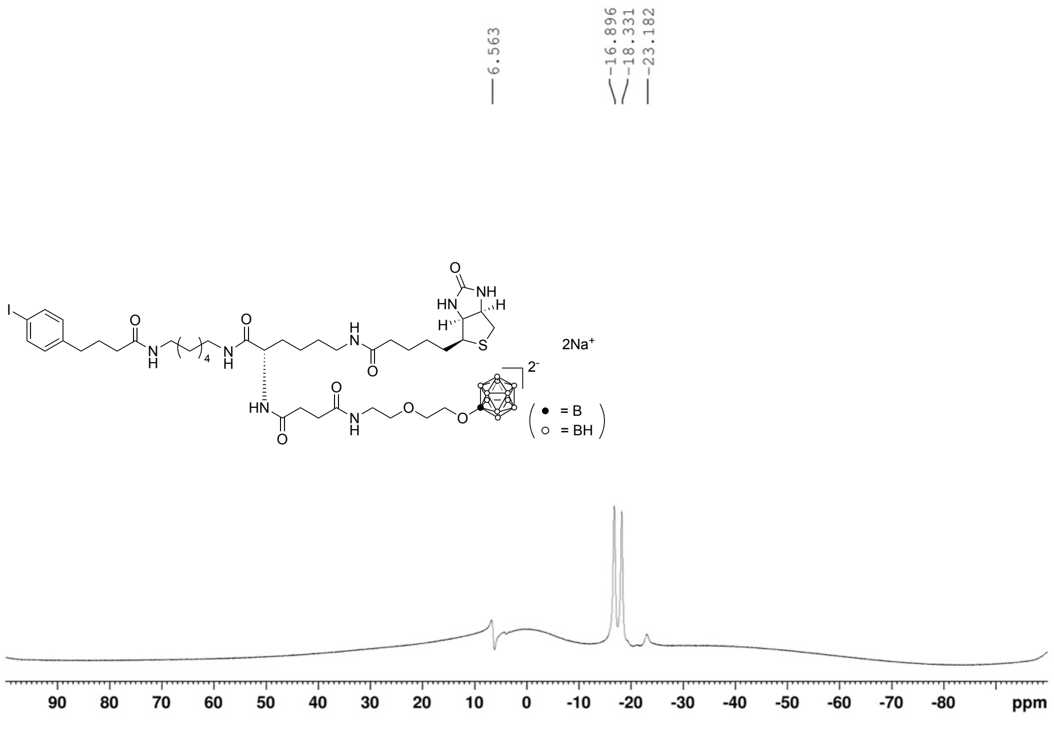

^{13}C NMR (125 MHz, DMSO-d_6)

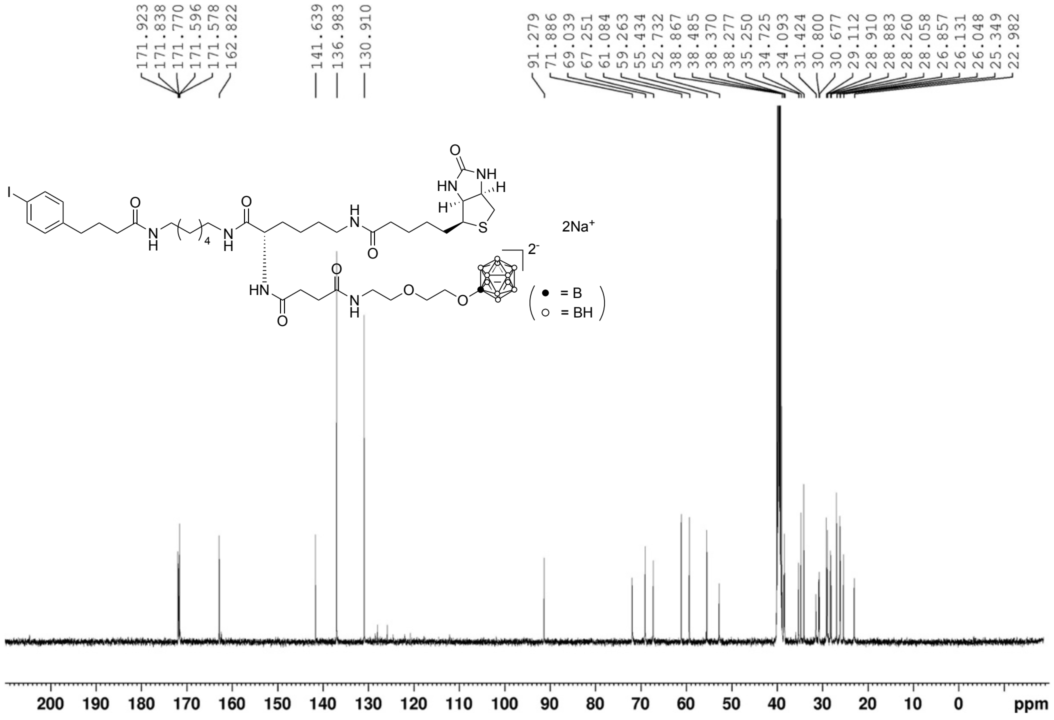

BBC

^{1}H NMR (500 MHz, D_2O)

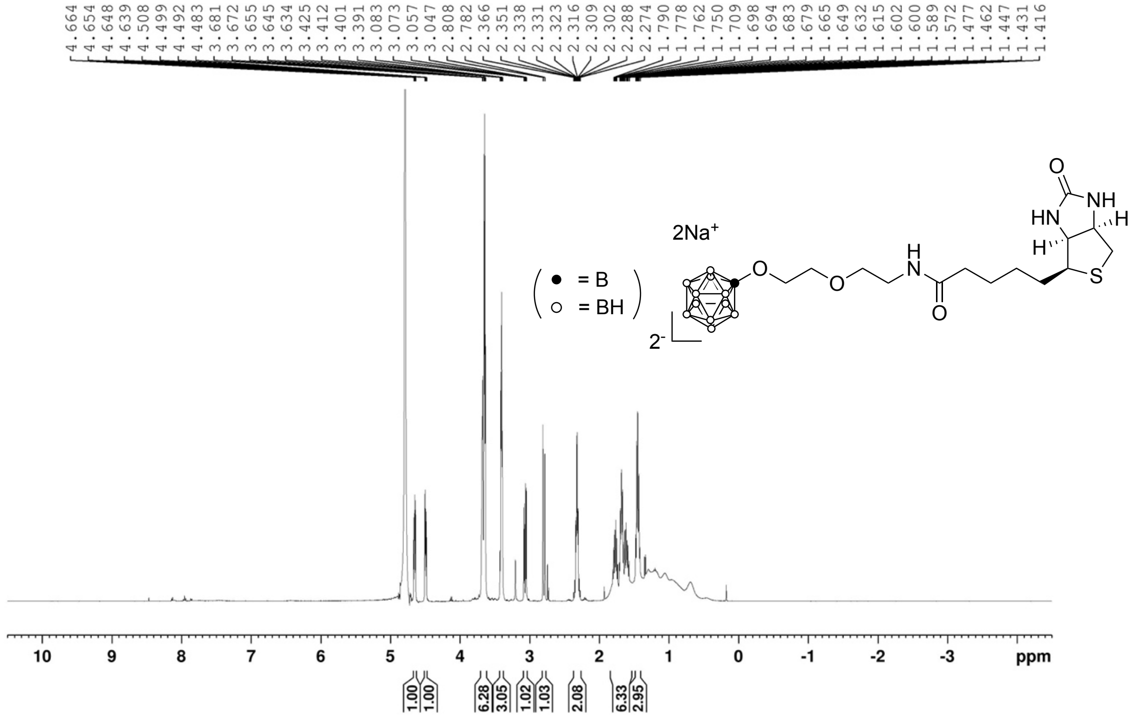

^{11}B NMR (160 MHz, D_2O)

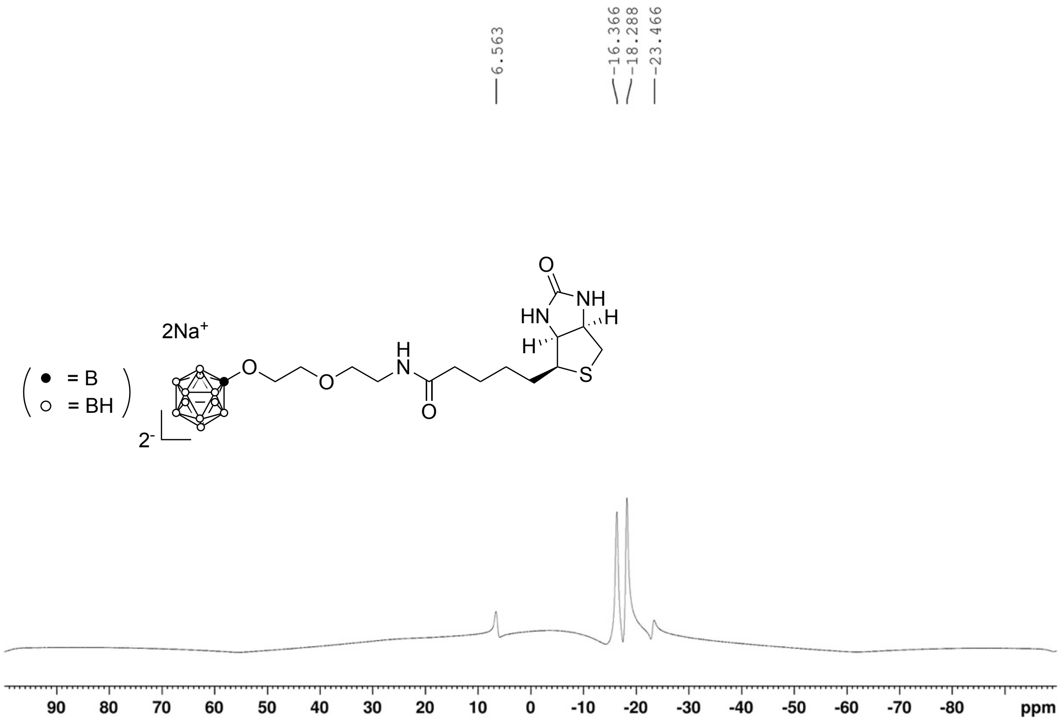

^{13}C NMR (125 MHz, D_2O)

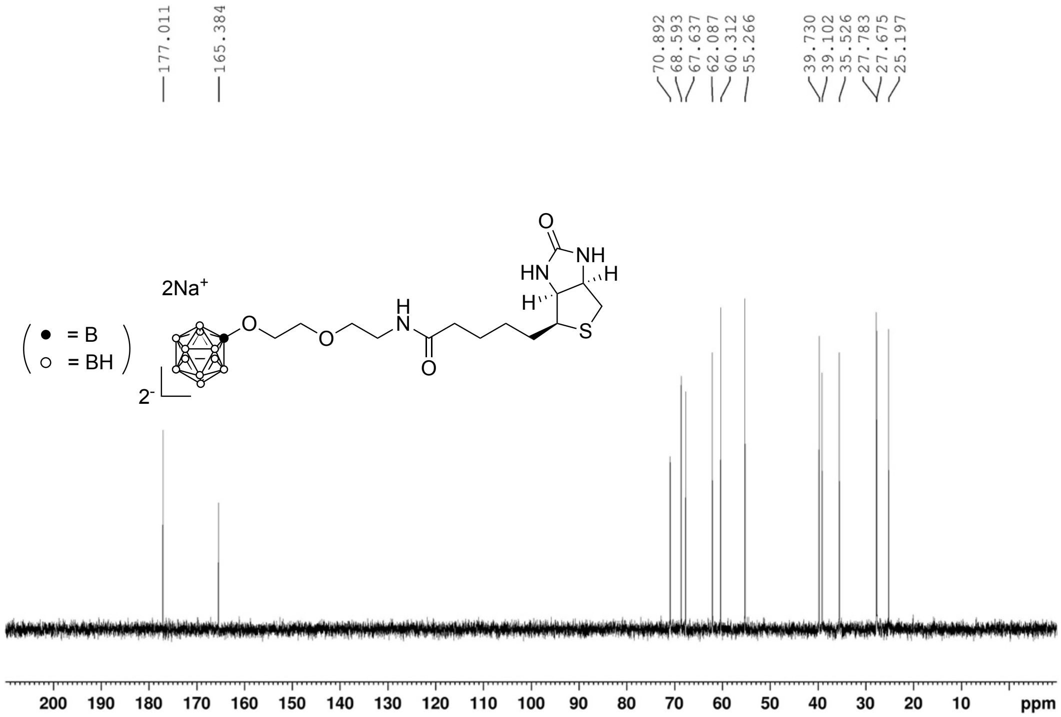

3. HPLC chromatograms

BBC-IP

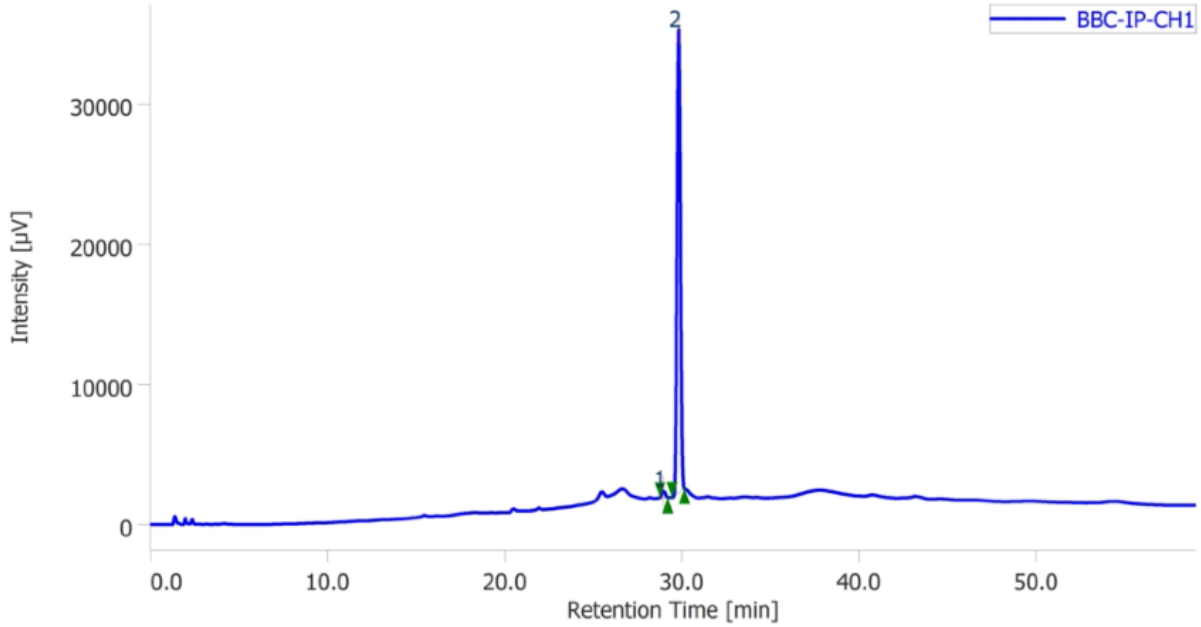

Peak Information

#	tR [min]	Area [µV·sec]	Height [µV]	Area [%]	Height [%]
1	28.958	6485	451	1.331	1.347
2	29.825	480674	32994	98.669	98.653

Analytical conditions: measurement wavelength, 254 nm; flow rate, 1.0 mL/min; eluent A, phosphate-buffered saline; eluent B, acetonitrile; gradient, 0-30 min, 0-40% B, 30-60 min, 40% B; column, JASCO, Finepak SIL C18S 5 µM, 4.6 × 150 mm.

BBC

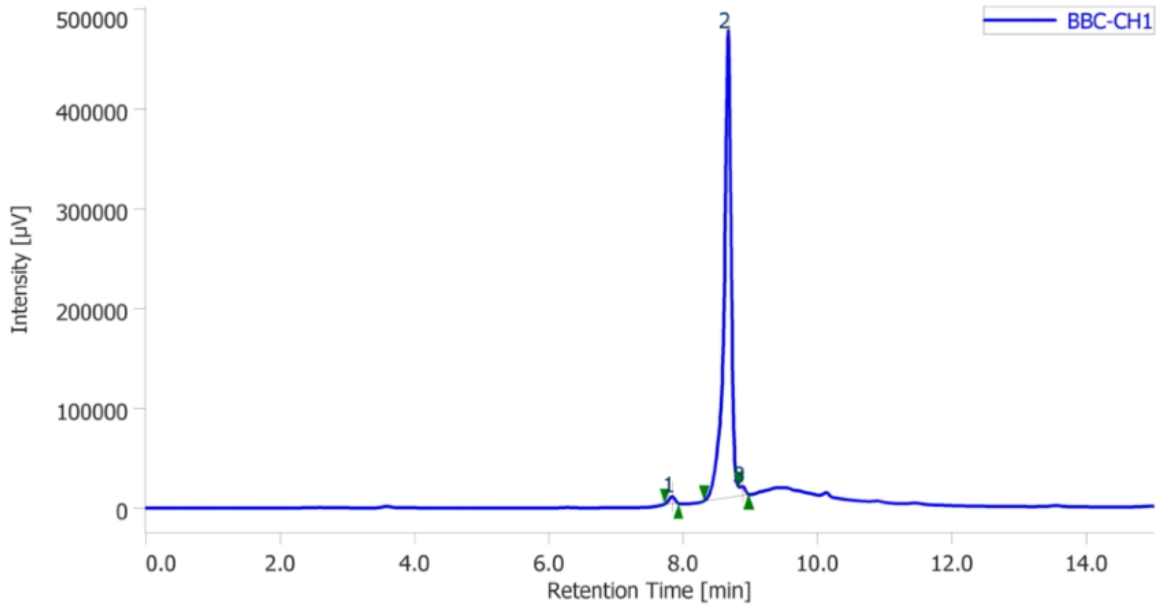

Peak Information

#	tR [min]	Area [μV·sec]	Height [μV]	Area [%]	Height [%]
1	7.833	44340	7337	1.201	1.534
2	8.667	3634643	467723	98.443	97.819
3	8.875	13152	3093	0.356	0.647

Analytical conditions: measurement wavelength, 254 nm; flow rate, 1.0 mL/min; eluent A, phosphate-buffered saline; eluent B, acetonitrile; gradient, 0-15 min, 0-20%; column, JASCO, Finepak SIL C18S 5 μM, 4.6 × 150 mm.
